# Spontaneous Haemoperitoneum in the Third Trimester of Pregnancy Due to Adenomyosis: A Case Report

**DOI:** 10.7759/cureus.63718

**Published:** 2024-07-02

**Authors:** Maja Krajec, Vojka Lebar, Lilijana Kornhauser Cerar, David Lukanovic, Leon Meglic

**Affiliations:** 1 Department of Gynaecological Oncology, Institute of Oncology Ljubljana, Ljubljana, SVN; 2 Department of Perinatology, Division of Gynaecology and Obstetrics, University Medical Centre Ljubljana, Ljubljana, SVN; 3 Department of Gynaecology, Division of Gynaecology and Obstetrics, University Medical Centre Ljubljana, Ljubljana, SVN

**Keywords:** acute abdomen, endometriosis, adenomyosis, pregnancy, haematoperitoneum

## Abstract

Spontaneous haemoperitoneum in pregnancy (SHiP) is a rare condition that can seriously endanger the life of both the mother and child. It can occur at any time during pregnancy but is most common in the last trimester. The etiology of SHiP is unknown. Endometriosis is one of the main risk factors for spontaneous haemoperitoneum due to the rupture of the utero-ovarian vasculature or bleeding from endometrial foci in the abdomen, but so is adenomyosis. We present an infrequent clinical case of a patient with uterine adenomyosis rupture and bleeding from endometrial foci in the third trimester of pregnancy.

## Introduction

Despite its rarity, spontaneous haemoperitoneum in pregnancy (SHiP) is an important clinical condition from the point of view of gynaecologists and obstetricians. It is a spontaneous, non-traumatic intraperitoneal haemorrhage during pregnancy or up to 42 days postpartum, with potentially many serious obstetric complications [1,2].

While SHiP can occur at any stage of pregnancy, it is most frequently observed in the third trimester, presenting as acute abdominal pain and requiring urgent surgical management [3].

Adenomyosis is a benign disorder where endometrial tissue is present within the myometrium. It is significantly associated with unfavourable perinatal outcomes. Women with adenomyosis often experience higher incidences of preterm delivery, preeclampsia (PE), cesarean delivery, postpartum haemorrhage, and delivering small-for-gestational-age infants [4]​.

## Case presentation

A 36-year-old primipara who conceived spontaneously and had gestational diabetes mellitus managed through dietary adjustments was admitted to the Department of Gynaecology, University Medical Centre Ljubljana, in Slovenia, due to acute abdomen at 26 weeks and three days of gestation. She was admitted to a peripheral hospital a few hours before this admission due to the sudden onset of severe abdominal pain. Initial laboratory tests revealed haemoglobin levels at 96 g/l (Table 1). An ultrasound was performed and showed a viable fetus with a heartbeat, adequate amniotic fluid, and placenta on the anterior side of the uterus extending beyond the internal uterine orifice, without evidence of retroplacental hematoma. Vaginal examination revealed a shortened, closed cervix, without discharge (Bishop’s score 3). The cardiotocograph was normal. The pregnant woman received spasm analgesic therapy: trospium chloride 0.2 mg and metamizole 2.5 g.

**Table 1:** Laboratory values before and after the surgery

Test	Unit	Reference range	On admission	After surgery	One day after surgery	Two days after surgery	Three days after surgery	Four days after surgery	Five days after surgery
White blood cells	10^9/L	4.0-10.0	/	21.9	13.5	7.7	10.6	7.6	9.8
Erythrocytes	10^12/L	4.2-5.4	/	4.08	3.29	3.54	3.14	2.8	2.85
Haemoglobin	g/L	120-160	96	126	98	106	96	84	85
C-reactive protein	mg/L	0-5.0	/	/	205	256	205	133	77
Procalcitonin	μg/L	0-0.50	/	0.08	1	/	0.93	0.55	0.29

The pregnancy was normal until 26 weeks and three days. The patient's medical history included three spontaneous pneumothoraces of unknown aetiology in one year prior to conception and a large loop excision of the transformation zone (LLETZ) of a pathological lesion on the cervix. We did not find any other medical conditions in the patient.

An abdominal ultrasound on admission showed a large amount of free fluid in the abdominal cavity and a viable pregnancy with no signs of labour. The abdomen was palpatory tense and painful. Due to developing hemorrhagic shock, the patient was immediately transferred to the operating theatre. In addition to the gynaecological surgeon and the obstetrician assistant, a neonatologist was also present. An abdominal surgeon also attended the operation because of a suspected perforation of a hollow organ.

**Figure 1:**
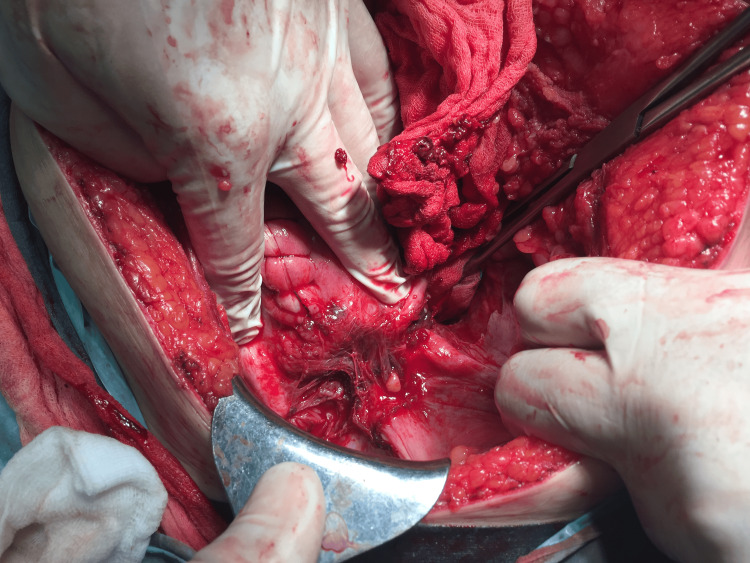
A large bleeding ulcer on the uterus to the left from the plica vesica uterina

On entering the abdominal cavity via a midline incision, approximately one litre of blood and clots were present in the abdomen. After examination of the abdominal cavity, gastrointestinal and vascular causes of bleeding were first excluded. In addition to a 5 cm large bleeding ulcer on the uterus to the left from the plica vesica uterina under the round ligament (Figure 1) and under the left adnexum, a ruptured left parametrium was present. Due to the life-threatening condition of the patient and the fetus, an emergency cesarean section with vertical incision was performed, delivering a breech-presenting female newborn weighing 820 g with an Apgar score of 3/6. The placenta appeared intact. After uterine exploration, no communication between the uterine cavity and the lesion above was found. Samples were taken for histological examination. After delivery and suturing of the uterus with a running suture, the patient underwent an abdominal hysterectomy without adnexa due to uncontrollable massive bleeding from the uterus, which was performed in a typical and uncomplicated manner. The final blood loss was estimated at 2200 ml. Intraoperatively, the cause of the bleeding was not identified. The patient received 1190 ml (which is four units) of blood group 0 positive erythrocyte transfusion with leucocytes removed. She recovered from the procedure without complications. She was discharged to home care on the ninth postoperative day. Figure 1 shows a large bleeding ulcer on the uterus to the left from the plica vesica uterina.

**Figure 2:**
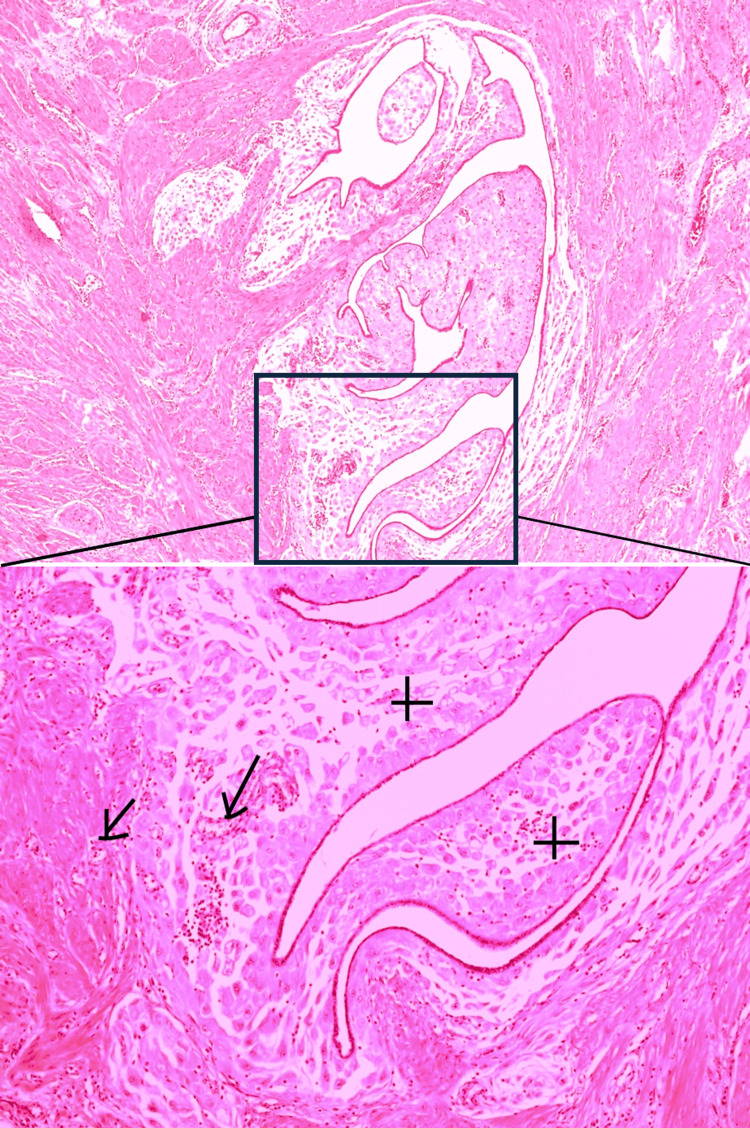
Decidual reaction of adenomyosis, which led to a uterine rupture There are abundant large polygonal and eosinophilic endometrial stromal cells (+) with small haemorrhages (arrows) in the subserosal area. Increased vascularity can also be observed due to hormonal influence and increased metabolic demands of the decidualized tissue. Areas of haemorrhage and hemosiderin-laden macrophages can sometimes be present due to bleeding from ectopic endometrial tissue. Findings are consistent with decidualized adenomyosis. Small magnification 40x, large 100x.

Uterine histology revealed adenomyosis with multiple, large foci overgrowing the serosa (Figure 2). Most of the foci were located in the subserosal myometrial area. Abundant decidualized endometriotic foci were demonstrated in the tissue samples taken from the uterine vesical fold and the peritoneum of the sigmoid colon.

The newborn was not breathing after birth, and due to low oxygen saturation (below 60% despite ventilation with a 100% oxygen mask), she was intubated three minutes after birth and transferred to the Neonatal Intensive Care Unit (NICU) of the Ljubljana Maternity Hospital with positive-pressure ventilation. Immediately after admission, she received a surfactant replacement, after which her oxygen and ventilatory support requirements rapidly decreased. Due to lung immaturity and complications from two attempts at extubation (swelling of the vocal cords), she required 23 days of synchronized intermittent positive-pressure ventilation support, followed by 19 days of non-invasive mask ventilation, and later low-flow supplemental oxygen via nasal tubes to achieve adequate oxygenation. During her stay in the NICU, she had hospital-acquired pneumonia caused by *Staphylococcus aureus* and was treated with flucloxacillin. To close the haemodynamically significant patent ductus arteriosus, a common pathology in such immature preterm infants, she received two courses of indomethacin. Because of anaemia, she required an early transfusion of concentrated red blood cells (on day 12), and later, recombinant human erythropoietin was administered to stimulate erythropoiesis. Despite laboratory signs of perinatal asphyxia (metabolic acidosis with pH 7.09 and BE of 12.2), there was no evidence of cerebral haemorrhage or asphyxia on ultrasound, and the girl's neurological status was considered normal for gestation until discharge. As a consequence of immaturity, the ophthalmologist diagnosed signs of stage 1 retinopathy of prematurity at 53 days of age (34 weeks postmenstrual age), which had regressed by the time of discharge. The girl was discharged home at 72 days of age (37 weeks postmenstrual age) weighing 2600 g, and still requiring supplemental oxygen due to chronic lung disease of immaturity.

The child was followed up in the outpatient clinic for children at risk until two years of corrected age. Oxygen supplementation was discontinued three months after discharge home. At the time of the check-ups, we initially noted a mild delay in gross motor skills, but at the last check-up, she had caught up with her peers in both growth and development.

## Discussion

Herein, we report a rare case of haemoperitoneum in pregnancy due to adenomyosis, presenting as acute abdominal pain in the third trimester. This condition led to significant intra-abdominal haemorrhage and necessitated immediate surgical intervention, including an emergency cesarean section and subsequent hysterectomy to control the bleeding.

The literature describes endometriosis and ovarian hyperstimulation during external assisted reproductive technology (IVF) as important risk factors for ShiP [3,5]. Pregnancies achieved via assisted reproductive technology and complicated with endometriosis can have a higher incidence and severity of SHiP than spontaneous pregnancies complicated with endometriosis [5]. A recent study by Mazzocco et al. has found that 0.3% of 362 pregnant women with endometriosis who conceived through assisted reproductive technology developed SHiP [6].

The ectopic decidua is an appearance of decidual tissue outside the uterus. The mechanism of occurrence is unknown [3,7]. Haematoperitoneum probably develops due to the transformation of the ectopic decidua of the preexistent endometriotic foci and invasion of the ectopic decidua into the uterine vasculature under the influence of endogenous progesterone, the level of which is high during pregnancy [2,8-10].

To date, isolated cases of intraperitoneal haemorrhage in pregnancy due to rupture of the utero-ovarian vasculature have been described. The pathogenesis of the occurrence is not clear. One possible cause is a physiological increase in blood flow during pregnancy, which subsequently causes dilatation of the vasculature and increases the chance of spontaneous rupture [10-12]. Spontaneous rupture of uterine arteriovenous malformations or fistulae is rare [13,14]. 

Differential diagnosis should exclude placental causes of bleeding, such as placental abruption, abnormally invasive placentation, and uterine scar rupture. A definitive diagnosis can only be made by laparotomy [3]. In our case, the cause of bleeding was not identified during the procedure. Histology showed adenomyosis with overgrowth of the serosal surface of the uterus causing an uncontrolled bleeding ulcer requiring hysterectomy. Most of the foci were located in the subserosal myometrial area. In individual peritoneal specimens, abundant endometriotic foci with decidua were demonstrated. The patient had a history of three spontaneous pneumothoraces of unclear aetiology. In consideration of the histological diagnosis, the patient may have concomitant endometriosis of the pleura.

Clinically, ShiP presents as an acute abdomen, with sudden onset of severe abdominal pain requiring immediate surgical intervention. The patient was in the third trimester of pregnancy with previously unknown endometriosis, which is consistent with the findings of Brosens et al. that ShiP is more common in the second and third trimesters of pregnancy and that endometriosis is the cause of bleeding in more than 50% of cases [3,5].

To the best of our knowledge from the reviewed cases, only two clinical cases of overgrowing adenomyosis causing uterine rupture and massive life-threatening bleeding in a pregnant woman have been described by now [15,16].

ShiP is a rare condition that can be life-threatening for both the mother and child. Maternal mortality due to ShiP has declined sharply over the years, to 4%, but fetal mortality remains high [3,17].

## Conclusions

Our case emphasizes the criticality of promptly recognizing and managing SHiP, a rare yet life-threatening condition. With endometriosis identified as a significant risk factor, understanding its pathophysiological mechanisms is paramount for effective intervention. Our findings, revealing uterine adenomyosis as a rare precipitant, highlight the complexity of SHiP aetiology. Despite challenges in intraoperative identification, our case points out the necessity of expedited surgical intervention to mitigate maternal and fetal risks. Continued research is imperative to elucidate SHiP's multifactorial aetiology and optimize clinical management strategies, ultimately improving outcomes for affected pregnant women and their infants.
